# PROTOCOL: Evidence‐informed practice versus evidence‐based practice educational interventions for improving knowledge, attitudes, understanding, and behavior toward the application of evidence into practice: A comprehensive systematic review of undergraduate students

**DOI:** 10.1002/cl2.1015

**Published:** 2019-07-23

**Authors:** Elizabeth Adjoa Kumah, Robert McSherry, Josette Bettany‐Saltikov, Sharon Hamilton, Julie Hogg, Vicki Whittaker, Paul van Schaik

**Affiliations:** ^1^ School of Health and Social Care Teesside University Middlesbrough UK; ^2^ School of Social Sciences, Humanities Law Teesside University Middlesbrough UK

Evidence‐informed practice versus evidence‐based practice educational interventions for improving knowledge, attitudes, understanding, and behavior toward the application of evidence into practice: A comprehensive systematic review of undergraduate health and social care students.

## 1 BACKGROUND

### 1.1 Description of the condition

Over the past three decades, there has been increasing attention on improving healthcare quality, reliability, and ultimately, patient outcomes, through the provision of healthcare that that is influenced by the best available evidence, and devoid of rituals and tradition (Andre, Aune, & Brænd, 2016; Melnyk, Gallagher‐Ford, Long, & Fineout‐Overholt, 2014; Sackett, Rosenberg, Gray, Haynes, & Richardson, 1996). There is an expectation by professional regulators such as the Nursing and Midwifery Council, United Kingdom (NMC, 2015) and the Health and Care Professions Council (HCPC, 2012) that the professional, as part of their accountability applies the best available evidence to inform their clinical decision‐making, roles and responsibilities. This is imperative for several reasons. Firstly, it enhances the delivery of healthcare and improves efficiency. Secondly, it produces better intervention outcomes and promotes transparency. Thirdly, it enhances co‐operation and knowledge sharing among professionals and service users, and ultimately, it improves patient outcomes and enhances job satisfaction. Indeed, the need to guide healthcare practice with evidence has been emphasized by several authors, including Kelly, Heath, Howick, & Greenhalgh, 2015; Nevo & Slonim‐Nevo, 2011; Scott & McSherry, 2009; Shlonsky & Stern, 2007; Smith & Rennie, 2014; Straus, Glasziou, Richardson, & Haynes, 2011; Tickle‐Degnen & Bedell, 2003; and Sackett et al., 1996. According to these authors, the effective and consistent application of evidence into healthcare practice helps practitioners to deliver the best care for their patients and patient relatives. Nevertheless, there is often an ineffective and inconsistent application of evidence into healthcare practice (McSherry, 2007; Melnyk, 2017; Nevo & Slonim‐Nevo, 2011).

**Figure 1:**
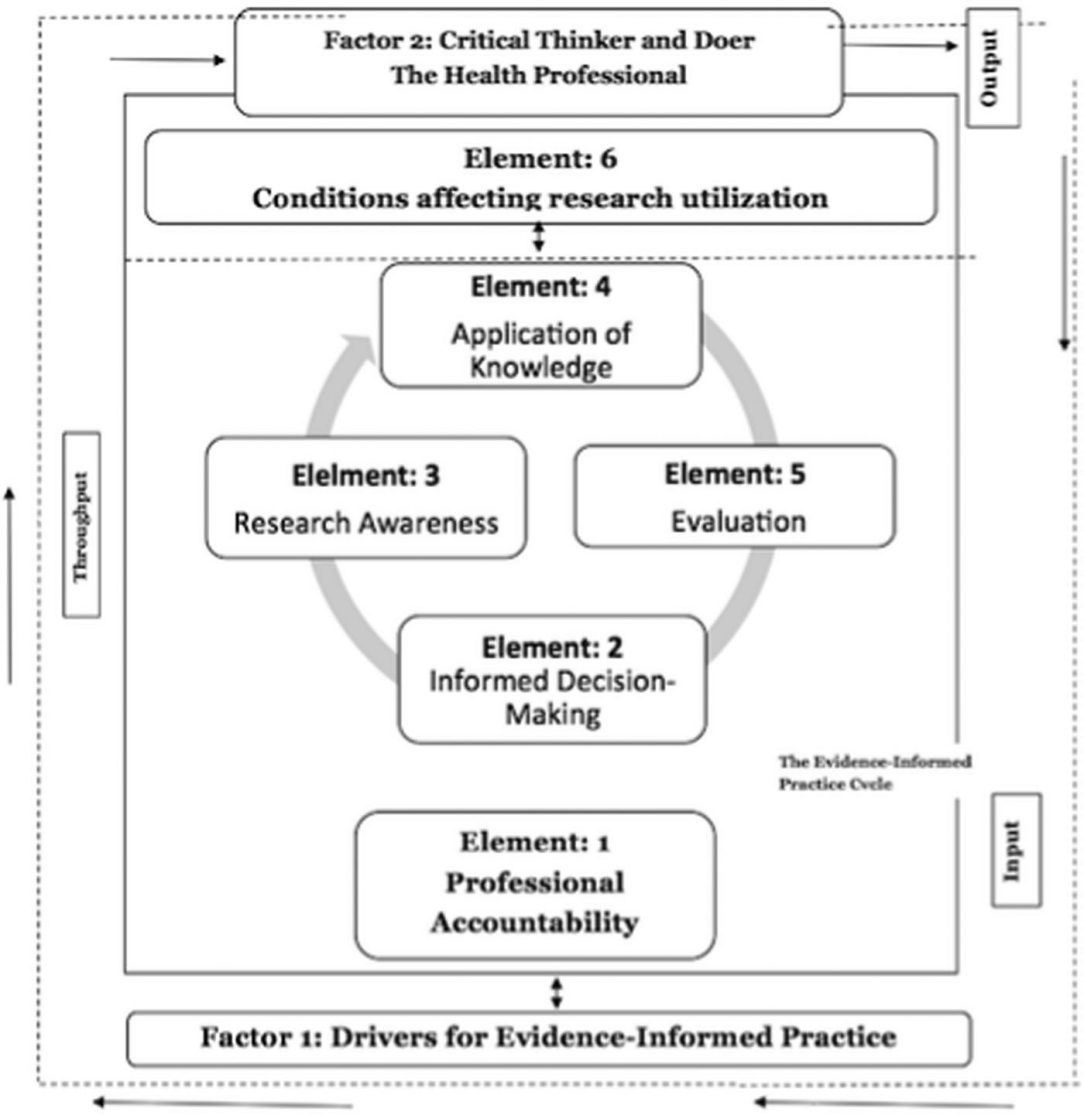
The evidence‐informed practice model

The two main concepts that have been associated with the application of evidence into healthcare practice are “evidence‐based practice” and “evidence‐informed practice”. Whilst Evidence‐based practice has been considered the gold standard for effective healthcare delivery, a large majority of healthcare practitioners continue to encounter multiple difficulties, which inhibit the rapid application of evidence into practice (Epstein, 2009; Glasziou, 2005; Greenhalgh, Howick, & Maskrey, 2014; McSherry, 2007; McSherry, Simmons, & Pearce, 2002; Melnyk, 2017; Nevo & Slonim‐Nevo, 2011). Nevo & Slonim‐Nevo, 2011 believe the application of evidence into practice should, in principle be “informed by” evidence and not necessarily “based on” evidence. This suggests that decision‐making in healthcare practice “might be enriched by prior research but not limited to it” (Epstein, 2009, p. 9). Similarly, McSherry, 2007 considers the application of evidence into practice (evidence‐informed practice) to be a systems‐based approach (i.e. made up of an input, throughput and an output), which contains, as part of its elements the principles of evidence‐based practice. McSherry, 2007 believes evidence‐based practice is the awareness, as well as the implementation of the relevant “research evidence” into practice. Hence, the author argues that the principles of evidence‐based practice are contained in the “research awareness” element of the “evidence‐informed practice model” (see Figure 1 for McSherry 2007 evidence‐informed practice model).

Currently, there is an on‐going debate in the literature as to which of these two concepts best facilitate the effective and consistent application of evidence into practice. Researchers, such as Melnyk 2017, Melnyk & Newhouse, 2014, and Gambrill, 2010 believe that knowledge and skills in evidence‐based practice help the healthcare professional to effectively apply evidence into practice. Conversely, Epstein, 2009; Nevo & Slonim‐Nevo, 2011; and McSherry, 2007 have argued the need to equip healthcare professionals with the necessary knowledge and skills of evidence‐informed practice in order to facilitate the effective and consistent application of evidence into practice. Moreover, whilst some authors, including Cardoso et al., 2017 and Glasziou, 2005 have used the two terms interchangeably, other researchers (such as Epstein, 2007; McSherry, 2007; Nevo & Slonim‐Nevo, 2011; and McSherry et al., 2002) have identified significant differences between the two concepts. These differences are described in the ensuing section.

It is imperative that healthcare training institutions produce graduates who are equipped with the knowledge and skills necessary for the effective and consistent application of evidence into practice (Dawes et al., 2005; Frenk et al., 2010; Melnyk, 2017). Hence, it is necessary for healthcare training institutions to include the principles involved in the application of evidence into practice, in undergraduate health and social care curricula. However, the question that arises is: which of the two concepts best facilitates the application of evidence into practice? While Melnyk, Fineout‐Overholt, Stillwell, & Williamson, 2010 have suggested a seven‐step approach to the application of evidence into practice (termed the “evidence‐based practice model”), McSherry, 2007 has argued that the principles involved in the application of evidence into practice is a systems‐based approach, with an input, throughput and an output (termed the “evidence‐informed practice model”).

The main purpose of this systematic review is to determine the differences and similarities, if any, between evidence‐informed practice and evidence‐based practice educational interventions; as well as the role each concept plays in the application of evidence into practice. In addition, the present systematic review aims to determine whether the two concepts act together, or individually to facilitate the effective application of evidence into practice. These aims will be achieved by exploring the effectiveness of evidence‐informed practice educational interventions versus evidence‐based practice educational interventions in improving the knowledge, attitudes, understanding, and behavior required for the effective application of evidence into practice among undergraduate pre‐registered health and social care students.

### 1.2 Description of the intervention

The gap between evidence and healthcare practice is well acknowledged (Lau et al., 2014; Melnyk 2017; Straus, Tetroe, & Graham, 2009). Difficulties in using evidence to make decisions in healthcare practice are evident across all groups of decision‐makers, including health care providers, policy makers, managers, informal caregivers, patients, and patient relatives (Straus et al., 2009). Consequently, several interventions have been developed to improve the implementation of evidence into healthcare practice and policy. Specifically, evidence‐based practice educational interventions are widely used and have been greatly evaluated (for example, Callister, Matsumura, Lookinland, Mangum, & Loucks, 2005; Dawley, Bloch, Suplee, McKeever, & Scherzer, 2011; Heye & Stevens 2009; Schoonees, Rohwer, & Young, 2017; and Goodfellow, 2004). Evidence‐informed practice educational interventions have also been used as well (for example, Almost et al., 2013), although to a much smaller extent. Conducting a systematic review of currently available research offers a rigorous process for evaluating the comparative effectiveness of both evidence‐informed practice and evidence‐based practice educational interventions.

Dawes et al., 2005 and Tilson et al., 2011 have each reported on Sicily statements, which have been made about the need for developing educational interventions on evidence‐based practice in healthcare. The statements were made separately in the “Evidence‐Based Healthcare Teachers and Developers” conference held in 2003 (Dawes et al., 2005) and 2009 (Tilson et al., 2011). The statements provide suggestions for evidence‐based practice competencies, curricula and evaluation tools for educational interventions. All health and social care students and professionals are required to understand the principles of evidence‐based practice, to have a desirable attitude towards evidence‐based practice and to effectively implement evidence‐based practice (Dawes et al., 2005). In order to incorporate a culture of evidence‐based practice among health and social care students, Melnyk, 2017 believes undergraduate health and social care research modules need to be based on the seven‐step model of evidence‐based practice that was developed by Melnyk et al., 2010. In addition, the curricula should include learning across the four components of evidence‐based practice, namely, knowledge, attitudes, behavior, and practice (Haggman‐Laitila, Mattila, & Melender, 2016).

Tilson et al., 2011 identified major principles for the design of evidence‐based practice evaluation tools for learners. Among the identified categories for evaluating evidence‐based practice educational interventions include the learner's knowledge of, and attitudes towards evidence‐based practice, the learner's reaction to the educational experience, behavior congruent with evidence‐based practice as part of patient care, and skills in implementing evidence‐based practice. The frameworks used in assessing the effectiveness of evidence‐based practice interventions need to reflect the aims of the research module. The aims also need to correspond to the needs and characteristics of learners. For example, students may be expected to perform the seven‐steps of evidence‐based practice, whiles health practitioners may be required to acquire skills in applying evidence into practice. In addition, the setting where learning, teaching and the application of evidence‐based practice occur must be considered Tilson et al., 2011.

Evidence‐informed practice, on the other hand, extends beyond the initial definitions of evidence‐based practice (LoBiondo‐Wood, Haber, Cameron, & Singh, 2013), and is more inclusive than evidence‐based practice (Epstein, 2009). This is due to the following reasons. Firstly, evidence‐informed practice recognises practitioners as critical thinkers and encourages them to be knowledgeable about findings from all types of research (including, systematic reviews, randomised controlled trials, qualitative research, quantitative research, and mixed methods), and to utilize it in an integrative manner. Secondly, evidence‐informed practice considers the best available research evidence, practitioner knowledge and experience, client preferences and values, and the clinical state and circumstances (Nevo & Slonim‐Nevo, 2011). However, Melnyk & Newhouse, 2014 (p. 347) disagreed with this assertion as a difference between the two concepts. According to the authors, like evidence‐informed practice, evidence‐based practice has broadened to "integrate the best evidence for well‐designed studies and evidence‐based theories (i.e., external evidence) with a clinician's expertise, which includes internal evidence gathered from a thorough patient assessment and patient data, and a patient's preferences and values". Although this statement may be true, the existing evidence‐based practice models (for example, DiCenso, Ciliska, & Cullum, 2005; Dufault 2004; Greenhalgh, Robert, & Bate, 2005; Melnyk et al 2010; Titler, Kleiber, & Steelman, 2001) place too much emphasis on “scientific evidence”, when making clinical decisions, and focus little or no attention to other forms of evidence such as the clinical context, patient values and preferences, and practitioner's knowledge and experiences (McTavish 2017; Miles & Loughlin, 2011).

Inasmuch as scientific evidence plays a major role in clinical decision‐making, the decision‐making process must be productive and adaptable enough to meet the on‐going changing condition and needs of the patient, as well as the knowledge and experiences of the health practitioner (LoBiondo‐Wood et al 2013; Nevo & Slonim‐Nevo, 2011). To this, researchers, including Nevo & Slonim‐Nevo, 2011 and McSherry 2007, have advocated for a creative and flexible model of applying evidence into practice, where healthcare practitioners are not limited to following a series of steps (as advocated in evidence‐based practice) in order to apply evidence into practice. Thirdly, unlike evidence‐informed practice, evidence‐based practice uses a formal hierarchy of evidence, which ranks certain forms of evidence (for example, systematic reviews and randomised controlled trials) higher than others (such as qualitative research and observational studies). Instead of the hierarchy of evidence, proponents of evidence‐informed practice support an integrative model of practice that considers all forms of studies and prefers the evidence that provides the best answer to the clinical question (Epstein, 2009). In place of the hierarchy of evidence, Epstein, 2011 suggested a “wheel of evidence,” where “all forms of research, information gathering, and interpretations would be critically assessed but equally valued” (p. 225). This is to ensure that all forms of evidence are considered during decision‐making in healthcare practice.

Evidence‐informed practice does not follow a stepwise approach to applying evidence into practice. Evidence‐informed practice is a systems‐based approach to applying evidence into practice, which comprises of input, throughput and an output (McSherry, 2007). McSherry, 2007 believes the actual process of applying evidence into practice occurs in a cyclical manner (termed the evidence‐informed cycle) and not stepwise. Evidence‐informed practice is adaptable and considers the complexities of health and healthcare delivery. Healthcare professionals live and work in a complex system. In fact, the clinical environment as well as health care delivery in itself is a complex system, made up of many interdependent parts (Sturmberg & Lanham, 2014). Hence, as previously stated, evidence‐informed practice considers several factors including, the culture and context of patient care, experiences of the healthcare professional, patient preferences and values, as well as factors that influence research utilization (such as workload, lack of organizational support, and time) in clinical decision‐making (LoBiondo‐Wood et al., 2013; McSherry, 2007; Nevo & Slonim‐Nevo, 2011). Thus, an evidence‐informed practice educational intervention needs to recognise the learner as a critical thinker who is expected to consider various types of evidence in clinical decision‐making (Almost et al., 2013; McSherry et al., 2002). One is not expected to be a researcher in order to effectively implement evidence‐informed practice. According to McSherry et al., 2002, the healthcare professional must be aware of the various types of evidence (such as the context of care, patient preferences, and experience, as well as clinician's skills and expertise), not just research evidence, in order to deliver person‐centred care. Table 1 presents a summary of the differences and similarities between evidence‐informed practice and evidence‐based practice.

**Table 1:** A summary of the differences and similarities between evidence‐informed practice and evidence‐based practice

Evidence‐based practice	Evidence‐informed practice	Similarities between evidence‐based practice and evidence‐informed practice
Evidence‐based practice adopts a “cookbook’ approach to applying evidence into practice, and so leaves no room for flexibility (Nevo & Slonim‐Nevo, 2011).	Evidence‐informed practice recognizes practitioners as critical thinkers (McSherry 2007; Nevo & Slonim‐Nevo, 2011), and encourages them to be creative and to consider the clinical state and circumstances when making patient care decisions.	Both evidence‐informed practice and evidence‐based practice are approaches for making informed clinical decisions (Woodbury & Kuhnke, 2014)
		Both evidence‐informed practice and evidence‐based practice integrate research with patient values and preferences and clinical knowledge and expertise (Melnyk & Newhouse, 2014)
The existing evidence‐based practice models (for example, DiCenso et al., 2005; Dufault, 2004; Greenhalgh et al., 2005; Melnyk et al 2010; Titler et al., 2001) rely heavily on scientific evidence, when making clinical decisions, and give little attention to other forms of evidence such as the clinical context, patient values and preferences, and practitioner's knowledge and experiences (McTavish, 2017; Miles & Loughlin, 2011)	The existing evidence‐informed practice models (for example, McSherry, 2007; Nevo & Slonim‐Nevo, 2011) are innovative and flexible. The client is at the centre, not the evidence (McTavish, 2017). One is not expected to be a researcher in order to effectively implement evidence‐informed practice; the healthcare professional must be aware of the various types of evidence, such as the context of care, patient preferences, and experience, as well as the clinician's skills and expertise, not just the research evidence, in order to deliver effective person‐centred care.	
Evidence‐based practice uses a formal hierarchy of evidence, which ranks certain forms of research evidence (for example, systematic reviews and randomized controlled trials) higher than others (such as qualitative research and observational studies).	Instead of the hierarchy of evidence, evidence‐informed practice supports an integrative model of practice that considers all forms of research evidence (including, systematic reviews, randomized controlled trials, qualitative research, quantitative research and mixed methods), and prefers the evidence that provides the best answer to the clinical question (Epstein, 2009).	
The existing models of Evidence‐based practice adopt a stepwise approach to applying evidence into healthcare practice.	Evidence‐informed practice is an integrative (McTavish, 2017) and a systems‐based approach to applying evidence into practice, which comprises of input, throughput and an output (McSherry, 2007)	
The linear approach of evidence‐based practice does not allow health practitioners to be creative enough, so as to meet the on‐going changing needs and conditions of the patient and the healthcare setting.	Evidence‐informed practice is adaptable, and considers the complexities of health and healthcare delivery (LoBiondo‐Wood et al., 2013; Nevo & Slonim‐Nevo, 2011). The evidence‐informed practice model considers several factors, such as the factors that influence research utilization (including workload, lack of organisational support, and time) in clinical decision‐making (McSherry, 2007).	

Table 1: For the purposes of this systematic review, the following operational definitions will apply:

*Evidence‐informed practice educational interventions* refer to any formal educational program that facilitates the application of the principles of the evidence‐informed practice model developed by McSherry, 2007. The evidence‐informed practice model (Figure 1), as developed by McSherry, 2007 is a systems‐based model comprising of an input (for example, roles and responsibilities of the health practitioner) throughput (i.e. research awareness, application of knowledge, informed decision‐making, evaluation) and an output, which is an empowered professional who is a critical thinker and doer (McSherry, 2007).

*Evidence‐based practice educational interventions* refer to any formal educational program that enhances the application of the principles of the evidence‐based practice model developed by Melnyk et al., 2010. The evidence‐based practice model developed by Melnyk et al., 2010 comprises of a seven‐step approach to the application of evidence into practice. These are (1) to cultivate a spirit of inquiry (2) ask a clinical question (3) search for the best evidence to answer the question (4) critically appraise the evidence (5) integrate the appraised evidence with own clinical expertise and patient preferences and values (6) evaluate the outcomes of the practice decisions or changes based on evidence and (7) disseminate evidence‐based practice results (Melnyk et al., 2010).

In this systematic review, it is not a requirement for eligible studies to mention specifically Melnyk et al., 2010's model of evidence‐based practice or McSherry, 2007's model of evidence‐informed practice as the basis for the development of their educational program. However, the content of the educational program in each of the studies to be included must include some, if not all, of the elements and/or principles of the aforementioned models.

In addition, definitions for “knowledge”, “attitudes”, “understanding” and “behavior” will be based on the Classification Rubric for Evidence‐based practice Assessment Tools in Education (CREATE) created by Tilson et al., 2011 as follows:

*Knowledge*: Knowledge refers to learners’ retention of facts and concepts about evidence‐informed practice and evidence‐based practice. Hence, assessments of evidence‐informed practice and evidence‐based practice knowledge might assess a learner's ability to define evidence‐based practice and evidence‐informed practice concepts, list their basic principles or describe levels of evidence.

*Attitudes*: attitudes refer to the values ascribed by the learner to the importance and usefulness of evidence‐informed practice and evidence‐based practice to inform clinical decision‐making.

*Understanding*: understanding refers to learners’ comprehension of facts and concepts about evidence‐based practice and evidence‐informed practice.

*Behavior*: Behavior refers to what learners actually do in practice. It is inclusive of all the processes that a learner would use in the implementation of evidence‐informed practice and evidence‐based practice, such as assessing patient circumstances, values, preferences, and goals along with identifying the learners’ own competence relative to the patient's needs in order to determine the focus of an answerable question.

The mode of delivery of the educational program could be in the form of workshops, seminars, conferences, journal clubs and lectures (both face‐to‐face and online). The content, manner of delivery and length of the educational program may differ in each of the studies to be included as there is no standard evidence‐informed practice/evidence‐based practice educational program.

In this systematic review, evidence‐informed practice and evidence‐based practice educational interventions that are targeted towards health and social care postgraduate students or registered health and social care practitioners will be excluded. Comparison interventions will include educational interventions that do not advance the teaching of the principles and processes of evidence‐informed practice and/or evidence‐based practice in healthcare or no intervention.

### 1.3 How the intervention might work

Most efforts to apply evidence into healthcare practice have either been unsuccessful or partially successful (Christie, Hamill, & Powers, 2012; Eccles, Grimshaw, Walker, Johnston, & Pitts, 2005; Grimshaw, Eccles, & Tetroe, 2004; Lechasseur, Lazure, & Guilbert, 2011; McTavish 2017). The resultant effects include ineffective patient outcomes, reduced patient safety, reduced job satisfaction, and increased staff turnover rate (Adams, 2009; Fielding & Briss 2006; Huston 2010; Knops, Vermeulen, Legemate, & Ubbink, 2009; Melnyk & Fineout‐Overholt, 2005; Schmidt & Brown, 2007). Hence, a lot of emphasis has been placed on teaching evidence‐based practice skills (Masters, 2009; Melnyk, 2017; Scherer & Smith, 2002; Straus, Ball, Balcombe, Sheldon, & McAlister, 2005) and/or evidence‐informed practice (Epstein, 2009; McSherry, 2007; McSherry et al., 2002; Nevo & Slonim‐Nevo, 2011) in undergraduate health and social care curricula. However, it remains unclear the exact components of an evidence‐based practice/evidence‐informed practice educational intervention. Consequently, healthcare instructors continue to encounter challenges when it comes to finding the most efficient approach to preparing health and social care students towards the application of evidence into practice (Almost et al., 2013; Flores‐Mateo & Argimon, 2007; Oh et al., 2010; Straus et al., 2005). This has resulted in an increase in the rate and number of research investigating educational interventions for enhancing knowledge, attitudes and skills towards, especially, evidence‐based practice (Phillips et al., 2013). There is also, empirical evidence (primary studies) to support a direct link between evidence‐based practice/evidence‐informed practice educational interventions and knowledge, attitudes, understanding and behavior, which in turn may affect the application of evidence into practice. However, participants in most of the studies reviewed were nursing students.

Ashtorab, Pashaeypoor, Rassouli, & Majd, 2014 developed an evidence‐based practice educational intervention for nursing students and assessed its effectiveness, based on Rogers’ diffusion of innovation model (Rogers, 2003). The authors concluded that evidence‐based practice education grounded on Roger's model leads to improved attitudes, knowledge and adoption of evidence‐based practice. According to the authors, Rogers’ diffusion of innovation model contains all the important steps that need to be applied in the teaching of evidence‐based practice.

Heye & Stevens, 2009 developed an evidence‐based practice educational intervention and assessed its effectiveness on seventy‐four (74) undergraduate nursing students using the ACE star model of knowledge transformation (Stevens, 2004). According to the authors, the Star model describes how evidence is progressively applied into healthcare practice by transforming the evidence through various stages (translation, integration, evaluation, discovery and summary). It was concluded that the students who participated in the educational program gained research appraisal skills and knowledge in the use of evidence in designing improvements in healthcare practice. In addition, the authors reported that undergraduate nursing students who were included in the study acquired evidence‐based practice competencies and skills that are required for the work environment.

Several other studies have reported on the effectiveness of evidence‐based practice educational interventions and their underpinning theoretical foundations: the Self‐directed learning strategies (Fernandez, Tran, & Ramjan, 2014; Kruszewski, Brough, & Killeen, 2009; Zhang, Zeng, Chen, & Li, 2012), the Constructivist Model of learning (Fernandez et al., 2014), Bandura's self‐efficacy theories (Kim, Brown, Fields, & Stichler, 2009) as well as the Iowa model of evidence‐based practice (Kruszewski et al., 2009). However, research in the area of evidence‐informed practice educational interventions has been limited. Almost et al., 2013 developed an educational intervention aimed at supporting nurses in the application of evidence‐informed practice. Prior to developing the intervention, the authors conducted interviews to examine the scope of practice, contextual setting and learning needs of participants. A Delphi survey was then conducted to rank learning needs, which were identified by the interview participants, in order to select the key priorities for the intervention. The authors then conducted a pre and post survey, before the intervention and six months after the intervention, respectively, to assess the impact of the intervention. Thus, the development of the intervention was learner‐directed, which reaffirms McSherry, 2007's description of the evidence‐informed practitioner as a critical thinker and doer. Unlike evidence‐based practice, practice knowledge and intervention decisions regarding evidence‐informed practice are enriched by prior research but not limited to it. In this way, evidence‐informed practice is more inclusive than evidence‐based practice (Epstein, 2009 p. 9). Nevo & Slonim‐Nevo, 2011 argue that rather than focusing educational interventions on the research‐evidence dominated steps of evidence‐based practice, research findings should be included in the intervention process, however, the process itself must be creative and flexible enough to meet the continually changing needs, conditions, experiences, and preferences of patients and health professionals.

**Figure 2:**
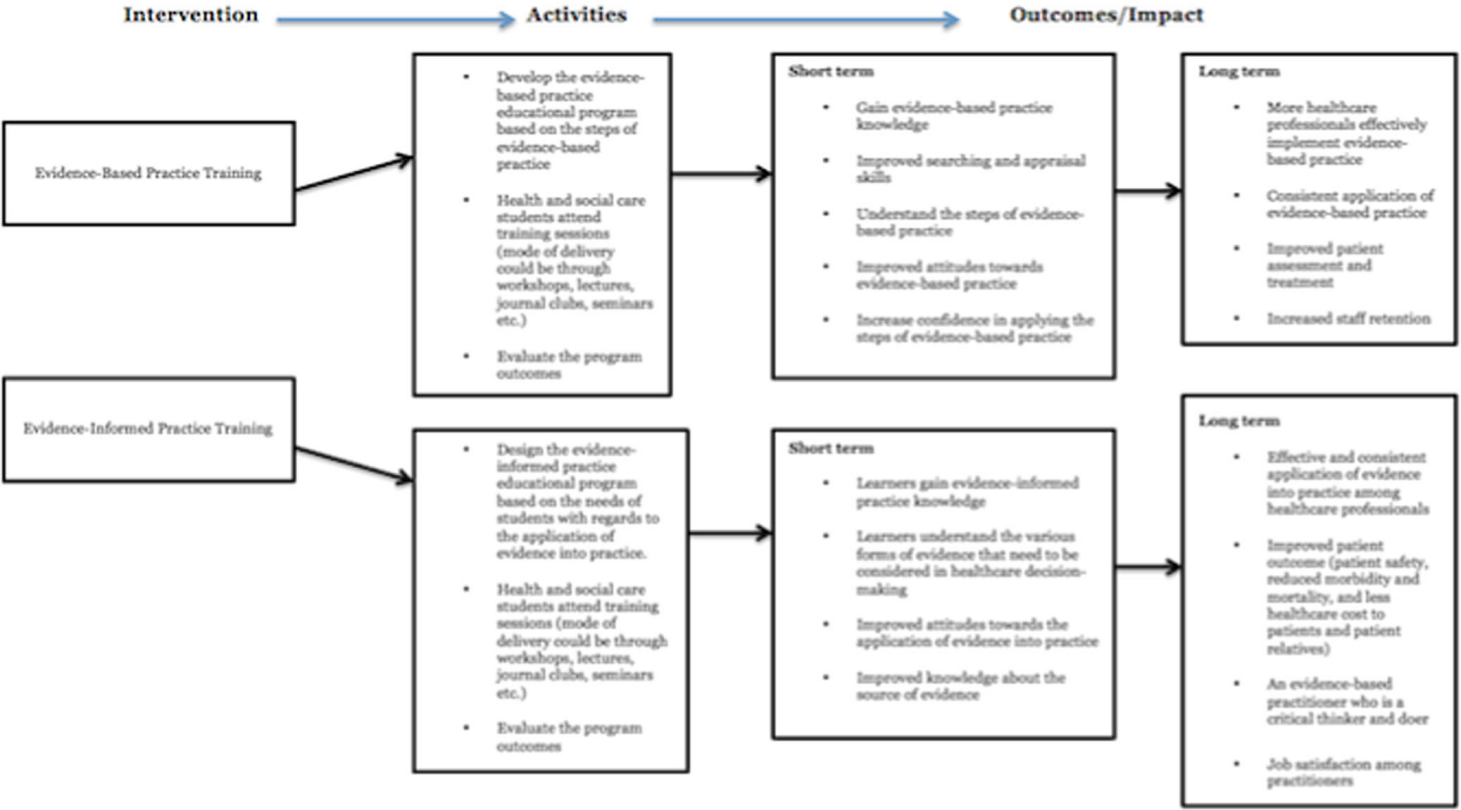
Logic model [Color figure can be viewed at wileyonlinelibrary.com]

A logic model has been presented in Figure 2 below to indicate the connection between evidence‐based practice/evidence‐informed practice educational intervention and outcomes.

### 1.4 Why it is important to do this review

Despite the seeming confusion surrounding the terms “evidence‐informed practice” and “evidence‐based practice” together with the on‐going debate in the literature as to which concept leads to better patient outcomes, no study, to the best of the researchers’ knowledge, has compared through a systematic review, the effects of the two concepts on the effective implementation of evidence into practice. A review of the literature reveals several systematic reviews conducted on evidence‐based practice educational interventions and the effects of such interventions. Examples of such systematic reviews are described below.

Young, Rohwer, Volmink, & Clarke, 2014 conducted an overview of systematic reviews that evaluated interventions for teaching evidence‐based practice to healthcare professionals (undergraduate students, interns, residents and practicing healthcare professionals). Comparison interventions in the study were no intervention or different strategies. The authors included 15 published and 1 unpublished systematic reviews. The outcome criteria included evidence‐based practice knowledge, critical appraisal skills, attitudes, practices, and health outcomes. In many of the included studies, however, the focus was on critical appraisal skills. The systematic reviews that were reviewed used a number of different educational interventions of varying formats (for example, lectures, online teaching and journal clubs), content and duration to teach the various component of evidence‐based practice in a range of settings. The results of the study indicated that multifaceted, clinically integrated interventions (for example, lectures, online teaching and journal clubs), with assessment, led to improved attitudes, knowledge, and skills towards evidence‐based practice. The majority of the included systematic reviews poorly reported poorly the findings from the source studies, without reference to significant tests or effect sizes. Besides, the outcome criteria (for example, knowledge, skills, attitudes, practices and health outcomes) were described narratively as improved or not, with the use of vote counting.

Coomarasamy & Khan, 2004 conducted a systematic review to evaluate the effects of standalone versus clinically integrated teaching in evidence‐based medicine on postgraduate healthcare students’ knowledge, critical appraisal skills, attitudes, and behavior. The results indicated that standalone teaching improved knowledge, but not skills, attitudes or behavior. Clinically integrated teaching, however, improved knowledge, skills, attitudes, and behavior. A similar systematic review by Flores‐Mateo & Argimon, 2007 identified a small significant improvement in postgraduate healthcare students’ skills, knowledge, behavior, and attitudes after participating in evidence‐based practice interventions. Furthermore, a systematic review of the literature has been conducted to identify the effectiveness of evidence‐based practice training programs and their components for allied health professionals (Dizon, Grimmer‐Somers, & Kumar, 2012). The researchers reported that irrespective of the allied health discipline, there was consistent evidence of significant changes in knowledge and skills among health practitioners, after participating in an evidence‐based practice educational program. In addition, recently, a systematic review has been conducted by Rohwer, Motaze, Rehfuess, & Young, 2017 to assess the effectiveness of the e‐learning of evidence‐based practice on increasing evidence‐based practice competencies in healthcare professionals (i.e. medical doctors, nurses, physiotherapists, physician assistants, and athletic trainers). The results indicated that pure e‐learning compared to no learning improved the knowledge of as well as the attitudes towards evidence‐based practice among the various professional groups.

Yet, according to a comprehensive literature review, no specific systematic review has been conducted on evidence‐informed practice educational interventions and the effects of such interventions on the knowledge, attitudes, understanding, and behavior of undergraduate health and social care students. Two reviews (namely, McCormack, Rycroft‐Malone, DeCorby, & Hutchinson, 2013; and Yost et al., 2015) conducted on evidence‐informed practice interventions focused on “change agency” and “knowledge translation” as interventions in improving evidence‐informed practice. For example, McCormack et al., 2013 conducted a realist review of strategies and interventions to promote evidence‐informed practice, but the authors focused only on “change agency” as an intervention aimed at improving the efficiency of the application of evidence. Also, a systematic review by Yost et al., 2015 concentrated on the effectiveness of knowledge translation on evidence‐informed decision‐making among nurses. A recent systematic review by Sarkies et al., 2017 focused on evaluating the effectiveness of research implementation strategies for promoting evidence‐informed policy and management decisions in healthcare. The authors also described factors that are perceived to be associated with effective strategies and the correlation between these factors. Nineteen papers (research articles) were included in the review. The results revealed a number of implementation strategies that can be used in promoting evidence‐informed policy and management in healthcare. The strategies included workshops, knowledge brokering, policy briefs, fellowship programs, consortia, literature reviews or rapid reviews, multi‐stakeholder policy dialogue, and multifaceted strategies. It is important to note that these strategies, though relevant, are more linked to healthcare management and policy decisions rather than typical patient care decision‐making/healthcare practice, which is the focus of the present systematic review.

The proposed systematic review offers originality and is significantly different from previously conducted systematic reviews on three fronts. Firstly, the present study focuses on pre‐registered undergraduate health and social care students as opposed to only nursing students, nurses or health care professionals. Secondly, the current study assesses the effectiveness of evidence‐informed practice educational interventions, while as recent studies by Rohwer et al., 2017 and Yost et al., 2015 assessed the effectiveness of e‐learning of evidence‐based health care and the effectiveness of knowledge translation on evidence‐informed decision‐making, respectively. Thirdly, the proposed systematic review focuses on comparing the effectiveness of evidence‐informed practice versus evidence‐based practice educational interventions on undergraduate health and social care students’ knowledge, attitudes, understanding, and behavior towards the application of evidence into practice. Furthermore, it determines whether evidence‐informed practice and evidence‐based practice act together, or individually to facilitate the application of evidence into practice.

It is imperative that a comprehensive systematic review of the literature that specifically compares the effectiveness of evidence‐informed practice versus evidence‐based practice educational interventions on undergraduate health and social care students is conducted. This will aid in reviewing and analysing current evidence‐informed practice and evidence‐based practice approaches in higher education settings. In addition, it is expected that the results of the current systematic review will help identify the impact of evidence‐informed practice as compared to evidence‐based practice educational interventions as well as gaps in the current literature. This is essential because it will offer direction for practice, policy and future inquiry in this growing area of research and practice.

## 2 OBJECTIVES

The primary objective of this systematic review is to evaluate and synthesize literature on the effectiveness of evidence‐informed practice versus evidence‐based practice educational interventions for improving knowledge, attitudes, understanding, and behavior of undergraduate health and social care students towards the application of evidence into practice.

Specifically, this systematic review will answer the following questions:

1. Is there a difference (i.e., difference in content, outcome) between evidence‐informed practice and evidence‐based practice educational interventions?
2. Does participating in evidence‐informed practice versus evidence‐based practice educational interventions facilitate the use of evidence in practice (for example, self‐reports on effective application of evidence into practice)?
3. Do both evidence‐informed practice and evidence‐based practice educational interventions targeted at undergraduate health and social care students affect patient outcomes (e.g. reduced morbidity and mortality, nosocomial infections)?
4. What factors affect the impact of evidence‐informed practice and evidence‐based practice educational interventions (e.g. course content, mode of delivery, multifaceted interventions, standalone intervention)?

## 3 METHODS

### 3.1 Criteria for considering studies for this review

#### 3.1.1 Types of studies

Based on the objectives and the outcome criteria for this systematic review, it is anticipated that relevant studies will have employed not only quantitative methodologies but also qualitative methods. Thus, this systematic review proposes to include both qualitative and quantitative research articles (a mixed‐methods systematic review).

Specifically, this systematic review will follow the recommended steps by Sandelowski, Leeman, Knafl, & Crandel, 2012: we will first conduct two separate syntheses for included quantitative and qualitative research. We propose to synthesize qualitative studies by way of meta‐aggregation and quantitative studies by way of meta‐analysis (Lockwood, Munn, & Porritt, 2015).

**Figure 3:**
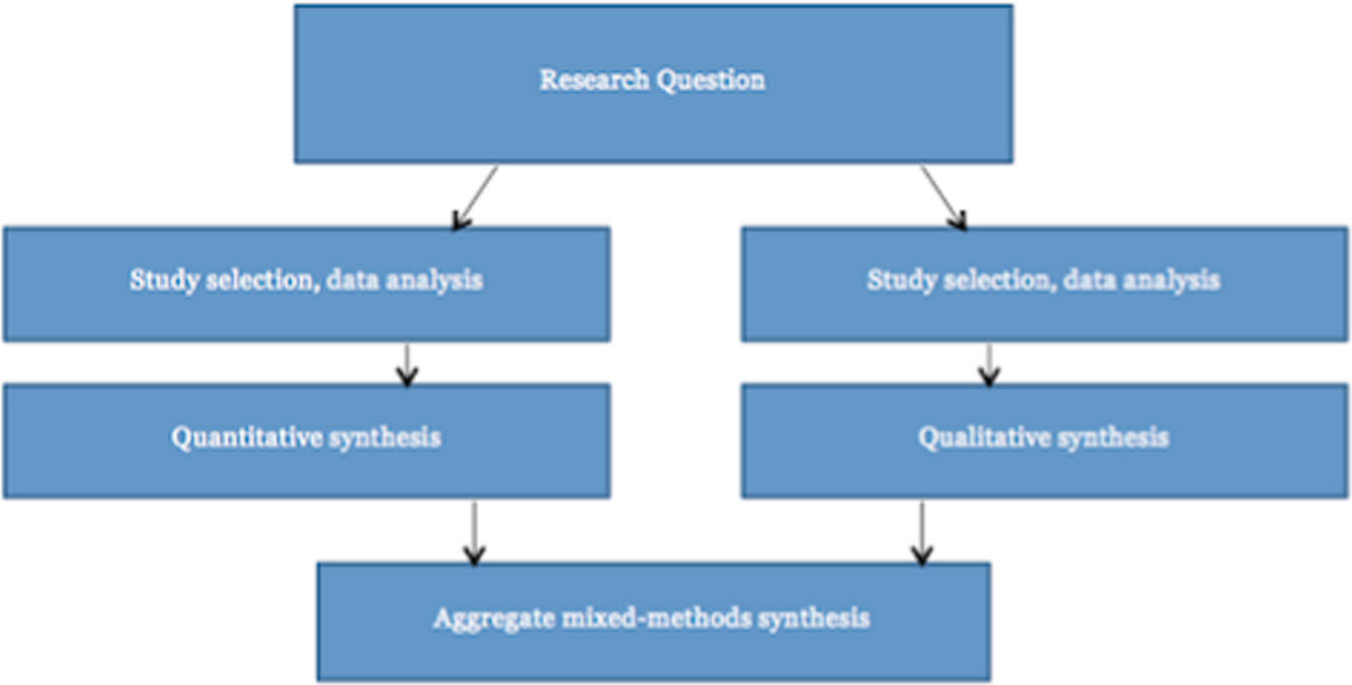
Summary of mixed methods strategy to be employed [Color figure can be viewed at wileyonlinelibrary.com]

We will then integrate the results of the two separate syntheses by means of an aggregative mixed‐methods synthesis. The two results (i.e. qualitative and quantitative results) will be integrated by translating findings from the quantitative synthesis into qualitative statements, by use of Bayesian conversion. This is because, it is preferable to translate quantitative synthesis into qualitative statements, than it is from qualitative to quantitative, as problems may arise when converting verbal accounts (such as few, many) into numbers or quantities (JBI 2014). Figure 3 presents the mixed‐methods approach to be employed in this systematic review.

To be eligible for inclusion in this review, study designs must meet at least one of the following criteria:

1. Randomized controlled trial: Studies in which participants are randomly assigned to intervention and comparison conditions.
2. Quasi‐randomised controlled trials: Studies where participants are assigned to intervention and comparison conditions through a quasi‐random approach, such as birth date, students identification number, or date of the week.
3. Quasi‐experimental controlled trial with individual level matching: Studies where participants in the intervention and comparison conditions are assigned to conditions in a non‐random manner (for example, study participants self‐select into groups). For such studies where participants in the intervention and comparison groups are not matched, there must be enough statistical information to enable us to evaluate pre‐test effect sizes for at least one outcome measure.
4. Quasi‐experimental controlled trial with pre‐test adjusted outcomes: Studies where the intervention and comparison conditions are assigned in a non‐random manner, but the pretest differences between groups have been adjusted by the study authors. Examples include pre‐test‐adjusted post‐test means and regression coefficients from models that adjust for pretest.
5. Quasi‐experimental controlled trial with pretest data: Studies where the intervention and comparison conditions are assigned in a non‐random manner, but pre‐test data are available for each outcome. For such studies, pre‐test data must be reported in a form that allows evaluation of the initial equivalence of the intervention and control groups on those variables through the calculation of effect size. For those outcomes with inapplicable pre‐test data, data for a close proxy of a pre‐test must be available.

Other studies to be eligible for inclusion include, before and after studies, prospective and retrospective cohort studies. In order to determine the effects of the educational intervention, the comparison groups that were used in included studies will be analysed. Additionally, primary studies that use descriptive epidemiological study designs will be eligible for inclusion in this systematic review. Examples of eligible epidemiological study designs will include case series, individual case reports, and descriptive cross‐sectional studies, focus groups, and interviews. Furthermore, other qualitative approaches such as ethnography, phenomenology, and grounded theory will be eligible for inclusion. Biases and limitations associated with any included study design will be discussed in relation to the impact it may have on the effectiveness of the intervention.

#### 3.1.2 Types of participants

To be eligible for inclusion in this systematic review, primary studies must include, as participants, undergraduate pre‐registered health and social care students in higher education (University) from any geographical area. Studies whose participants, are registered health and social care practitioners and postgraduate students will be excluded from this review.

#### 3.1.3 Types of interventions

In this systematic review, we will include studies that evaluate any formal evidence‐based practice and/or evidence‐informed practice educational interventions aimed at improving undergraduate pre‐registered health and social care students’ knowledge, attitudes, understanding and behavior in the application of evidence into healthcare practice. These two interventions will then be compared to determine whether the two concepts act together or individually to facilitate the application of evidence into practice. If it emerges that the two concepts act individually, we will determine which of them better facilitates the effective application of evidence into practice.

As mentioned above, in this systematic review, an evidence‐informed practice educational intervention refers to any formal educational program that facilitates the application of but not limited to the evidence‐informed practice model described by McSherry, 2007. The evidence‐informed practice model, as developed by McSherry, 2007 is a systems‐based model comprising of an input (for example, roles and responsibilities of the health practitioner) throughput (i.e., research awareness, application of knowledge, informed decision‐making, evaluation) and an output, which is an empowered professional who is a critical thinker and doer (McSherry, 2007). Conversely, in this systematic review, an evidence‐based practice educational intervention refers to any formal educational program that enhances the application of evidence‐based practice models. An example of such a model is the evidence‐based practice model developed by Melnyk et al., 2010. The evidence‐based practice model developed by Melnyk et al., 2010 comprises of a seven‐step approach to the application of evidence into practice. It is not a requirement for included studies to mention specifically Melnyk et al., 2010's model of evidence‐based practice or McSherry, 2007's model of evidence‐informed practice as the basis for the development of their educational program. However, the content of the educational program in each of the studies to be included must include some, if not all, of the elements and/or principles of the aforementioned models.

The mode of delivery of the educational programs may be in the form of workshops, seminars, conferences, journal clubs and lectures (both face‐to‐face and online). The content, manner of delivery and length of the educational program may differ in each of the studies to be included as there is no standard evidence‐informed practice/evidence‐based practice educational program.

Evidence‐informed practice and evidence‐based practice educational interventions that are targeted at health and social care postgraduate students or registered health and social care practitioners will be excluded. Comparison conditions will include educational interventions that do not advance the teaching of the principles and processes of evidence‐informed practice and/or evidence‐based practice in healthcare.

#### 3.1.4 Types of outcome measures

##### Primary outcomes

In this systematic review, studies will be eligible for inclusion if they address at least one of the following constructs as an outcome:

1. Participants’ knowledge about evidence‐informed practice and/or evidence‐based practice.
2. Participants’ understanding of evidence‐informed practice and/or evidence‐based practice.
3. Participants’ attitudes towards evidence‐informed practice and/or evidence‐based practice.
4. Participants’ behavior towards evidence‐informed practice and evidence‐based practice.

As previously stated, the above constructs (Knowledge, attitudes, understanding, and behavior) will be defined based on the Classification Rubric for Evidence‐based practice Assessment Tools in Education (CREATE), developed by Tilson et al., 2011:

*Knowledge*: Knowledge will refer to learners’ retention of facts and concepts about evidence‐informed practice and evidence‐based practice. Hence, assessment of evidence‐informed practice and evidence‐based practice knowledge might assess a learner's ability to define evidence‐based practice and evidence‐informed practice concepts, list their basic principles or describe the levels of evidence.

*Attitudes*: attitudes will refer to the values ascribed by the learner to the importance and usefulness of evidence‐informed practice and evidence‐based practice to inform clinical decision‐making.

*Understanding*: understanding will refer to learners’ comprehension of the facts and concepts relating to evidence‐based practice and evidence‐informed practice.

*Behavior*: Behavior will refer to what learners actually do in practice. It is inclusive of all the processes that a learner uses in the application of evidence‐informed practice and evidence‐based practice, such as assessing the scientific evidence, patient circumstances, values, preferences, and goals along with identifying the learners’ own competence relative to the patient's needs in order to determine the focus of an answerable question.

Measurement of the above outcomes may be conducted using standardized or unstandardized instruments. This is because, to the researchers’ knowledge, there is no uniform tool for evaluating evidence‐based practice and evidence‐informed practice educational interventions. Specific measures will include, but not be limited to:

1. The use of a standardized questionnaire to evaluate knowledge, attitude, understanding, and behavior towards the application of evidence into practice. Examples of such questionnaires include, but are not limited to the Evidence‐Based Practice Belief (EBPB) Scale and Evidence‐Based Practice Implementation (EBPI) scales developed by Melnyk, Fineout‐Overholt, & May, 2008. The EBPB scale is a 16‐item questionnaire that allows measurement of an individual's belief about the values of evidence‐based practice and the ability to implement evidence‐based practice, whereas the EBPI scale is an 18‐item questionnaire that evaluates the extent to which evidence‐based practice is implemented.
2. Examples of unstandardized instruments include, but are not limited to self‐reports from participants and researcher administered measures.

##### Secondary outcomes

Studies that measure the impact of evidence‐informed practice and/or evidence‐based practice educational programs on patient outcomes will be included.

Examples of patient outcome indicators to be assessed include; user experience, length of hospital stay, nosocomial infections, patient and health practitioner satisfaction, mortality, and morbidity rates.

###### Duration of follow‐up

In the current systematic review, no limit will be placed on the duration of the follow‐up. The rationale is to give room for studies with either short or long term follow‐up duration to be eligible for inclusion.

###### Types of settings

This systematic review will include primary studies from any geographical area. However, due to language translation issues, only studies written in English will be included. Studies whose title and abstracts are in English and meet the inclusion criteria, but the full article is reported in another language will be included subject to the availability of translation services.

###### Time

To qualify for inclusion in the current systematic review, studies must have been published during the period from 1996 (the date when evidence‐based practice first emerged in the literature) (Closs & Cheater, 1999; Sackett et al., 1996), to the date when the literature search will be conducted.

### 3.2 Search methods for identification of studies

#### 3.2.1 Search terms and keywords

We will use a combination of keywords and terms related to the population, intervention, outcome, and study design to conduct the search. Specific strategies for each database will be explored, such as the use of Boolean operators (for example, OR, AND), wildcards (such as?), phrase operators (e.g., ""), and truncations (including *). This will be done in order to ensure search precision and sensitivity. In addition, our search strategy will have three sets of terms: the population, intervention(s) and outcomes. We will use limiting commands to narrow the results by dates, language, and type of study design. Below are examples of anticipated search terms to be used:

1. *Targeted population*: nurs* OR physio* OR “occupa* therap*” OR “dental Hygiene” OR “undergraduate healthcare student*" OR "undergraduate social care student*” OR baccalaureat* OR “social work” OR dent* OR BSc OR student* OR “higher education” OR “undergrad* nurs* student*”
2. *Intervention*: evidence‐informed* OR evidence‐based* OR “evidence‐informed practice” OR “evidence‐based practice” OR EBP OR EIP OR “evidence‐informed practice education” OR “evidence‐based practice education” OR “evidence into practice” OR evidence‐informed near. practice teaching learning OR evidence‐based near. practice teaching learning
3. *Outcomes*: “knowledge, attitudes, understanding and behavio* regarding EBP” OR “knowledge near. attitudes understanding behavio* regarding EIP OR “Knowledge of evidence‐informed*” OR “knowledge of evidence‐based*” OR “patient outcome*” OR outcome*
4. *Study design/type*: trial* OR “randomi?ed control trial” OR “qua?i‐experiment*” OR random OR experiment OR “control* group*” OR program OR intervention OR evaluat* OR qualitative OR quantitative OR ethnograpy OR "control* study" OR "control* studies" OR "control* design*" OR "control* trial*" OR "control group design" OR RCT OR rct OR "trial registration"

#### 3.2.2 Management of references

We will import the full set of search results directly into an Endnote Library. Where this is not possible, we will manually enter search results into the Endnote Library. An Endnote library will make it easier to identify duplicates and to manage references.

#### 3.2.3 Search Strategy

The current systematic review will utilize six strategies, in order to identify published and unpublished studies that meet the inclusion criteria described above. These strategies have been outlined below.

##### Electronic searches

1. Electronic Database Search
  - Academic search complete
  - Academic search premier
  - AMED
  - Australian education index
  - British education index
  - Campbell systematic reviews
  - Canada bibliographic database (CBCA Education)
  - CINAHL
  - Cochrane Library
  - Database of Abstracts of Reviews on Effectiveness
  - Dissertation Abstracts International
  - Education Abstracts
  - Education complete
  - Education full text: Wilson
  - ERIC
  - Evidence‐based program database
  - JBI database of systematic reviews
  - Medline
  - PsycInfo
  - Pubmed
  - SciELO (Scientific Electronic Library Online)
  - Scopus

2. A web search using search engines
  - Google
  - Google scholar

3. Grey literature search
  - OpenGrey (System for Information on Grey Literature in Europe)
  - System for information on Grey Literature
  - The Society for Research on Educational Effectiveness
  - Virginia Henderson Global Nursing e‐Repository

Appendix 3 illustrates the search strategy for the MEDLINE database searched on the EBSCOhost platform. We will modify the search terms and strategies for the different databases.

##### Searching other resources

4. Hand searching. The table of content of at least three journals that published most of the studies identified as eligible for inclusion will be hand searched for additional relevant studies. Examples include Worldviews on Evidence‐Based Nursing Journal, British Medical Journal, and the British Journal of Social Work.
5. Track bibliographies of previously retrieved studies and literature reviews. The reference list of previously conducted systematic reviews, meta‐analysis and primary studies will be screened for other relevant studies.
6. Contact leading authors. The Corresponding authors of identified eligible abstracts, whose full texts are unavailable will be contacted to request for full‐text reports.

### 3.3 Data collection and analysis

#### 3.3.1 Selection of studies

The search output will be screened by two independent authors (either EAK and JBS or SH and RM) for relevant studies. The title and abstract of search output will first be screened, followed by the full text of articles with a seemingly relevant abstract. These articles will be assessed for eligibility using the pre‐specified inclusion and exclusion criteria. Studies that meet the inclusion criteria will be assessed independently by two authors (either SH and RM or EAK and JBS) for methodological validity using standardized critical appraisal instruments from the Joanna Briggs Institute Meta‐Analysis of Statistics Assessment and Review Instrument (JBI‐MAStARI). This includes, but not limited to, the JBI‐MAStARI checklist for case‐control studies, checklist for case reports, checklist for cohort studies, checklist for quasi‐experimental, checklist for randomised controlled trials, and checklist for analytical cross‐sectional studies. Any disagreements that may arise between authors will be resolved through discussion, if no agreement can be reached, a third author will be consulted.

#### 3.3.2 Data extraction and management

Data will be extracted from included papers using standardized data extraction tools from JBI‐MAStARI. Information to be extracted from quantitative studies will include study design, interventions, population, outcomes of significance to the review questions, and specific objectives. See Appendix 1 for the quantitative data extraction form. While this form is currently generic, we will include information specific to this systematic review, such as methods used to assess the impact of evidence‐informed practice/evidence‐based practice educational interventions on patient outcomes.

#### 3.3.3 Assessment of risk of bias in included studies

For each included study, two authors (either RM and SH or EAK and JBS) will independently assess the risk of bias. This will be done using the Cochrane Collaboration's Risk of Bias tool (Higgins & Green 2011). Discrepancies between reviewers will be resolved through discussion and consultation with a third author (VW). Studies will be categorized as having a high, low, or unclear risk of bias. The following criteria will be used to assess the risk of bias:

##### Random sequence generation

Studies will be categorized as having a high risk of bias if the authors used a non‐random sequence generation process, for example, the sequence generated by the preference of the study participants, even or odd date of birth, or availability of the intervention. Studies will be judged as having a low risk of bias if a random sequence generation process was used, and the process used in generating the allocation sequence is described in sufficient detail and able to produce comparable groups.

##### Allocation concealment

Studies will be deemed as having a low risk of bias if the method used in generating the allocation sequence was adequately concealed from study participants, such that study participants are unable to foresee group allocation. Studies will be judged as having a high risk of bias if the process used in generating allocation sequence was open such that study participants are able to predict group allocation. This introduces selection bias. An example includes using a list of random numbers.

##### Blinding of participants and personnel

Inadequate blinding results in participants and personnel having different expectations for their performance, hence biasing the results of the trial. Studies will be considered as having a low risk of bias if participants and trial personnel are blind to allocation status.

##### Blinding of outcomes assessors

We will examine included studies to determine if outcome assessors were blind to allocation status. Studies will be considered as having a low risk of bias if outcomes are assessed by independent investigators who had no previous knowledge of group allocation.

##### Incomplete outcome data

We will assess studies to determine if there are any missing outcome data. We will examine the differences between intervention and control groups in relation to measurement attrition and the reasons for missing data. Studies with low attrition (<20%), no attrition, or no evidence of differential attrition will be considered as having a low risk of bias. Use of Intention to Treat (ITT) analysis and methods of account for missing data (for example, using missing multiple imputations) will be recorded.

##### Selective outcome reporting

We will assess studies for reporting bias to determine whether there are inconsistencies between measured outcomes and reported outcomes. Studies will be considered as having a low risk of bias if the results section of publications clearly show that all pre‐specified outcomes are reported.

#### 3.3.4 Measures of treatment effect

##### Continuous data

For continuous data, where outcomes on the same scale are presented, we will use mean difference, with 95% confidence interval. However, where outcome measures differ between studies, we will use the standardized mean difference as the effect size metric based on Hedges’ g, which is calculated using the formula below:

SMD = Difference in mean outcome between groups ⁄ Standard deviation of outcome among participants.

##### Dichotomous data

For dichotomous data, we will calculate the risk ratio (and its 95% confidence interval) for the occurrence of an event. For the purpose of meta‐analysis, we will convert risk ratios to the standardized mean difference, by the use of David Wilson's practical effect size calculator.

Meta‐regression will be used to assess the impact of moderator variables on the intervention effect size. Moderator analysis will be conducted if a reasonable number of eligible research articles are identified and if the required data is presented in the report.

##### Studies with multiple groups

For studies with one control group versus two or more intervention groups, and all the interventions are regarded as relevant to the study, the following options will be used: 1) if the intervention groups are not similar, the sample size of the control group will be divided into two (or more based on the number of intervention groups) and will then be compared with the intervention groups 2) if the intervention groups are similar, the two groups will be treated as a single group. Therefore, two effect size estimates will be provided in this study. This is to ensure that participants in the control group are not “double counted” (Higgins & Green, 2011). We will employ a similar approach, but in reverse, in the event that an included study has one intervention group but two control groups. Additionally, if an included study contains an irrelevant and relevant intervention group, we will only include data from the relevant intervention group for analysis

#### 3.3.5 Unit of analysis issues

In this systematic review, it is anticipated that included studies may have either involved individual participants or clusters (groups) of participants as units of analysis. In the event that cluster‐randomised trials (i.e. studies where participants are allocated as a group rather than as individuals) are identified as eligible, we will use standard conversion criteria as recommended in the Cochrane Handbook (Higgins & Green, 2011). This will be done only if such studies have not been properly adjusted for clustering (for example, by the use of multi‐level modeling or robust standard errors).

The Cochrane Handbook (Higgins & Green, 2011) recommends guidelines to be followed in calculating the effective sample size in a cluster‐randomised trial. According to the Handbook, the effective sample size can be calculated by dividing the original sample size by the design effect. This equals 1+(M‐1)*ICC, where *M* is the average cluster size and ICC is the Intra‐cluster Correlation Coefficient.

#### 3.3.6 Dealing with missing data

Missing data for each of the included studies will be reported. We will contact the first author of studies with an incomplete report on data to request relevant information that is missing from the report.

If requested data is not provided, our options for dealing with missing data will be based on whether data is “missing at random” or “missing not at random.” If data is missing at random (that is, if the fact that they are missing is unrelated to actual values of the missing data), data analysis will be conducted based on the available data.

However, if data is missing not at random (that is, if the fact that they are missing is related to the actual missing data), we will impute the missing data with replacement values, and treat these values as if they were observed (for example, last observation carried forward, imputing an assumed outcome such as assuming all were poor outcomes, imputing the mean, imputing based on predicted values from a regression analysis).

#### 3.3.7 Assessment of heterogeneity

Heterogeneity will be assessed through the comparison of factors such as participant demographics, type of intervention, type of control comparators and outcome measures. Heterogeneity will be assessed and reported visually and by examining the I² statistic, which describes the approximate proportion of variation that is due to heterogeneity rather than sampling error. This will be supplemented by the Chi² test, where a P value < 0.05 indicates heterogeneity of intervention effects. In addition, we will estimate and present Tau², along with its CIs, as an estimate of the magnitude of variation between studies. This will provide an estimate of the amount of between‐study variation.

#### 3.3.8 Assessment of reporting biases

We will assess studies for reporting bias to determine whether there are inconsistencies between measured outcomes and reported outcomes. Studies will be considered as having a low risk of bias if the results section of publications clearly show that all pre‐specified outcomes are reported.

#### 3.3.9 Data synthesis

Narrative and statistical methods will be used to synthesise included studies. Data synthesis will be focused on calculating the effect sizes of the included studies. We will conduct meta‐analysis if our search yields sufficient (i.e. 2 or more) eligible studies that can be grouped together satisfactorily. A logical approach will be used when combining studies in meta‐analysis. Decisions on combining studies in meta‐analysis will be based on two reasons: 1) a sufficient number of eligible studies with similar characteristics 2) similar characteristics shared by those eligible studies may include the type of intervention and the targeted outcome of the intervention. Where a meta‐analysis is being conducted, we will employ the Comprehensive Meta‐analysis Software developed by Borentein, Hedges, Higgins, & Rothstein, 2005. We will conduct separate analyses for primary outcomes (i.e. knowledge, attitudes, behavior, and understanding) and secondary outcomes (i.e. patient outcome). In addition, separate analyses will be conducted for the effect of evidence‐based practice and evidence‐informed practice interventions. The evidence‐based practice versus evidence‐informed practice interventions comparisons will be determined by conducting a mean comparison test between the two concepts. The intervention versus control comparisons for each of the concepts will be based on adjusted post‐test means which control for imbalance at pre‐test. If this information is not available, the pre‐test mean effect size will be subtracted from the post‐test mean effect size. We will use the unadjusted pooled standard deviation.

#### 3.3.10 Subgroup analysis and investigation of heterogeneity

Heterogeneity will be assessed through the comparison of factors such as participant demographics, type of intervention, type of control comparators and outcome measures. Heterogeneity will be assessed and reported visually and by examining the I² statistic, which describes the approximate proportion of variation that is due to heterogeneity rather than sampling error. This will be supplemented by the Chi² test, where a P value < 0.05 indicates heterogeneity of intervention effects. In addition, we will estimate and present Tau², along with its CIs, as an estimate of the magnitude of variation between studies. This will provide an estimate of the amount of between‐study variation. Sensitivity and subgroup analyses will also be used to investigate possible sources of heterogeneity.

#### 3.3.11 Sensitivity analysis

Sensitivity analysis will be conducted to determine whether the overall results of data analysis are influenced by the removal of:

1. Unpublished studies
2. Studies with outlier effect sizes
3. Studies with a high risk of bias
4. Studies with missing information (for example, incomplete presentation of findings

##### Treatment of qualitative research

###### Assessment of methodological quality of qualitative papers

Included qualitative studies will be assessed independently by two authors (either SH and RM or EAK and JBS) for methodological validity using the JBI Qualitative Assessment and Review Instrument (JBI‐QARI). Any disagreements that may arise between authors will be resolved through discussion, if no agreement could be reached, a third author will be consulted.

##### Data extraction and management

Data will be extracted from included papers using standardized data extraction tool, namely, the JBI‐QARI. Information to be extracted from qualitative studies will include population, study methods, details about the phenomena of interest, outcomes of significance to the review question and specific objectives. See Appendix 2 for the qualitative data extraction form. While this form is currently generic, we will include information specific to this systematic review, such as methods used to assess the impact of evidence‐informed practice/evidence‐based practice educational interventions on patient outcomes.

##### Data synthesis and analysis

Where possible, qualitative research findings will be pooled using JBI‐QARI. This will involve the synthesis or aggregation of findings to generate a set of statements that represent that aggregation. Findings will be assembled based on their quality, and also, by grouping findings with similar meanings together. We will then perform a meta‐synthesis of these groups or categories so as to produce a single set of comprehensive synthesized findings. In the event that textual pooling is not possible, we will present findings in narrative form.

Finally, results from both the quantitative review and the qualitative review will be integrated using the JBI Mixed Methods Aggregation Instrument (MMARI). The integration will be achieved by translating findings from the quantitative review into qualitative results through the use of Bayesian conversion to generate synthesized results.

## 4 RESULTS

### 4.1 Description of studies

**Results of the search**

**Included studies**

**Excluded studies**

### 4.2 Risk of bias in included studies

**Allocation (selection bias)**

**Blinding (performance bias and detection bias)**

**Incomplete outcome data (attrition bias)**

**Selective reporting (reporting bias)**

**Other potential sources of bias**

## Effects of interventions

**Effects of interventions**

## 5 DISCUSSION

**Summary of main results**

**Overall completeness and applicability of evidence**

**Quality of the evidence**

**Potential biases in the review process**

**Agreements and disagreements with other studies or reviews**

## AUTHORS’ CONCLUSIONS

**Implications for practice**

**Implications for research**

## CONTRIBUTIONS OF AUTHORS

1. Content and Systematic Review methodology: Ms. Elizabeth Adjoa Kumah is a registered general nurse who has worked mainly in the critical care setting as a nurse supervisor and patient advocate. She has been actively engaged in teaching healthcare students in the clinical setting and serving as a mentor. She is currently pursuing a Ph.D. Health program, with evidence‐informed practice educational interventions as the area of research focus. She brings knowledge about the content both in terms of teaching healthcare students about the application of research evidence into practice and theoretically for improving knowledge of evidence‐informed practice and how it enhances evidence‐based practice skills, attitude and behavior in the educational setting. Elizabeth is passionate about improving the standard of patient care and patient outcome, which she believes could be achieved by the effective and consistent implementation of evidence‐informed practice. She will also help with the methodological aspects of the systematic review.
2. Content and Systematic review methods: Professor Robert McSherry will bring both methodological as well as content expertise relating to evidence‐informed practice and the development of teaching programs to the team. His area of expertise is around the evidence‐informed practice, patient safety, quality and clinical governance using practice development. Practice development is about promoting person‐centred care and approaches, which Rob has integrated effectively within both educational and research programs. He is the co‐author of a book on systematic reviews and has over thirty years’ experience as a registered general nurse. Robs educational and professional expertise has been recognized and rewarded internationally and nationally. He was awarded the highly prestigious National Teaching Fellow award in the UK in 2011.
3. Content and systematic review methods: Dr. Josette Bettany‐Saltikov will bring significant expertise in Systematic review methods and content to this systematic review, both in terms of knowledge about evidence‐based practice and knowledge about developing educational programs. She has taught systematic review methods to university students at all levels for over 15 years. She has also published a book on how to conduct a systematic review and has been involved in three Cochrane reviews, one of which she led. She has authored a number of systematic reviews on diverse topics published in other journals and has significant experience of developing educational programmes from her teaching experience as a university Senior lecturer for 23 years.
4. Content and systematic review methods: Professor Sharon Hamilton will bring expertise in systematic reviewing. She is the director of the Teesside Centre for Evidence‐Informed Practice: A Joanna Briggs Institute Centre of Excellence, and has conducted a number of qualitative and quantitative reviews. Sharon is a registered nurse and has research expertise in the evaluation of clinical interventions.
5. Information retrieval: Mrs. Julie Hogg brings Information retrieval expertise to the team. Julie is an Academic Librarian at Teesside University and will carry out a thorough and systematic search of the literature.
6. Statistical analysis: Mrs. Vicki Whittaker is a very experienced statistician with over 18 years of experience in teaching and advising students and academics on their research projects and clinical trials. She has been involved in data analysis and meta‐analysis of numerous research projects and systematic reviews.

## DECLARATIONS OF INTEREST

The review team declares no potential conflicts of interest.

### DIFFERENCES BETWEEN PROTOCOL AND REVIEW Published notes

**Characteristics of studies**

**Characteristics of included studies**

**Characteristics of excluded studies**

**Characteristics of studies awaiting classification**

**Characteristics of ongoing studies**

### SUMMARY OF FINDINGS TABLES

## ADDITIONAL TABLES

## REFERENCES TO STUDIES

### Included studies

**Excluded studies**

**Studies awaiting classification**

**Ongoing studies**

## OTHER REFERENCES

### Additional references

## SOURCES OF SUPPORT

### Additional references

- Teesside University, UK

This review forms part of a Ph.D. programme, which is supported and funded by Teesside University, Middlesbrough.

### External sources

- No sources of support provided

**Feedback**

## Appendix

**1 Quantitative Data Extraction form**

JBI‐MAStARI

**2 Qualitative Data Extraction Form**

JBI‐QUARI

**3 MEDLINE Search Strategy**

**Table:**

#	Query
S76	S17 AND S35 AND S51 AND S75
S75	S52 OR S53 OR S54 OR S55 OR S56 OR S57 OR S58 OR S59 OR S60 OR S61 OR S62 OR S63 OR S64 OR S65 OR S66 OR S67 OR S68 OR S69 OR S70 OR S71 OR S72 OR S73 OR S74
S74	"mixed method*"
S73	trial*
S72	"epidemiological stud*"
S71	"before and after stud*"
S70	"retrospective stud*"
S69	"prospective stud*"
S68	"descriptive stud*"
S67	"grounded theory"
S66	phenomenolog*
S65	interview*
S64	"focus group"
S63	"cross sectional"
S62	"case series"
S61	"cohort stud*"
S60	ethnography
S59	"qualitative stud*"
S58	experiment*
S57	"trial registration"
S56	"control group design"
S55	"control* trial*"
S54	"control* design*"
S53	randomi?ed control* trial* or rtc* or random* control* trial*
S52	random*
S51	S36 OR S37 OR S38 OR S39 OR S40 OR S41 OR S42 OR S43 OR S44 OR S45 OR S46 OR S47 OR S48 OR S49 OR S50
S50	"patient experience*"
S49	"quality of care"
S48	"patient outcome*"
S47	"professional responsibilit*"
S46	"professional accountability"
S45	"informed decision‐making"
S44	"research implement*"
S43	"research aware*"
S42	"application of evidence‐informed*"
S41	"application of evidence‐based*"
S40	"application of knowledge*"
S39	understand*
S38	behavio*
S37	attitude*
S36	knowledge*
S35	S18 OR S19 OR S20 OR S21 OR S22 OR S23 OR S24 OR S25 OR S26 OR S27 OR S28 OR S29 OR S30 OR S31 OR S32 OR S33 OR S34
S34	lecture*
S33	course*
S32	"education* program*"
S31	"education* intervention"
S30	evidence‐based practice vurses evidence‐informed practice
S29	compar* evidence‐based practice and evidence‐informed practice
S28	"evidence‐informed practice teaching and learning"
S27	"evidence‐informed practice teaching and learning"
S26	"evidence‐based practice teaching and learning"
S25	"evidence‐informed practice education"
S24	"evidence‐based practice education"
S23	ebp
S22	eip
S21	evidence‐informed*
S20	evidence‐based*
S19	"evidence‐informed practice"
S18	"evidence‐based practice"
S17	S1 OR S2 OR S3 OR S4 OR S5 OR S6 OR S7 OR S8 OR S9 OR S10 OR S11 OR S12 OR S13 OR S14 OR S15 OR S16
S16	undergraduate student*
S15	baccalaureate
S14	undergraduate social care student*
S13	undergraduate healthcare student*
S12	dental therap* student*
S11	dental hygiene student*
S10	(MH "Students, Dental")
S9	paramedic student*
S8	social work student*
S7	radiography student*
S6	student* midwi*
S5	occupation* therap* student*
S4	(MH "Students, Health Occupations")
S3	student* physical therap*
S2	student* physiotherap*
S1	(MH "Student* Nurs*")
